# Spike-timing prediction in cortical neurons with active dendrites

**DOI:** 10.3389/fncom.2014.00090

**Published:** 2014-08-13

**Authors:** Richard Naud, Brice Bathellier, Wulfram Gerstner

**Affiliations:** ^1^Department of Physics, University of OttawaOttawa, ON, Canada; ^2^Cortical Dynamics and Multisensory Processing Team, Unit of Neuroscience Information and Complexity, CNRS UPR-3239Gif-sur-Yvette, France; ^3^School of Computer and Communication Sciences and School of Life Sciences, Ecole Polytechnique Federale de LausanneLausanne, Switzerland

**Keywords:** dendrites, neuron models, cortical neurons, spike train analysis, models, theoretical

## Abstract

A complete single-neuron model must correctly reproduce the firing of spikes and bursts. We present a study of a simplified model of deep pyramidal cells of the cortex with active dendrites. We hypothesized that we can model the soma and its apical dendrite with only two compartments, without significant loss in the accuracy of spike-timing predictions. The model is based on experimentally measurable impulse-response functions, which transfer the effect of current injected in one compartment to current reaching the other. Each compartment was modeled with a pair of non-linear differential equations and a small number of parameters that approximate the Hodgkin-and-Huxley equations. The predictive power of this model was tested on electrophysiological experiments where noisy current was injected in both the soma and the apical dendrite simultaneously. We conclude that a simple two-compartment model can predict spike times of pyramidal cells stimulated in the soma and dendrites simultaneously. Our results support that regenerating activity in the apical dendritic is required to properly account for the dynamics of layer 5 pyramidal cells under *in-vivo*-like conditions.

## 1. Introduction

Partially neglected for a long time, dendrites have been recently shown to treat synaptic input in a surprising variety of modes (Stuart et al., 2007). Indeed, experiments have revealed that dendrites are excitable and that they can generate either sodium (Golding and Spruston, 1988), NMDA (Schiller et al., 2000) or calcium (Llinas and Sugimori, 1980) spikes. One particularly striking example is found in pyramidal cells of deep cortical layers. In these cells, a coincidence between a back-propagating action potential and dendritic input can trigger voltage-sensitive ion channels situated on the apical dendrite more than 300 μm from the soma (Larkum et al., 1999, 2001). The somatic membrane potential increases only after the activation of dendritic ion channels. This often resulting in a burst of action potentials. Bursts in these cells can therefore signal a coincidence of input from the soma (down) with inputs in the apical dendrites (top). Such top-down coincidence detection is one computation that is attributed to dendritic processes. Other allegedly dendritic computations include subtraction (Gabbiani et al., 2002), direction selectivity (Taylor et al., 2000), temporal sequence discrimination (Branco et al., 2010), binocular disparity (Archie and Mel, 2000), gain modulation (Larkum et al., 2004) and self-organization of neuron networks (Legenstein and Maass, 2011). These computations rely on the dendrite acting as an excitable subunit (Polsky et al., 2004; Stuart et al., 2007).

Models of large pyramidal neurons with active apical dendrites were first described by Traub et al. (1991) for the hippocampus. This model of the large CA3 pyramidal neurons included voltage-dependent conductances on the dendrites. It is a model based on the Hodgkin-Huxley description of ion channels. Cable properties of dendrites are taken into account by segmenting the dendrite into smaller compartments. The resulting set of equations is solved numerically. A simplified version of this model was advanced by Pinsky and Rinzel (1994). They have reduced the model to a dendritic compartment and a somatic compartment connected by an effective conductance. The model has a restricted set of five ion channels and accounts for bursting of CA3 pyramidal cells.

Models specific to deep cortical cells have been described by extending the approach of Traub et al. (1991); Schaefer et al. (2003) used morphological reconstruction to define compartments. This model could reproduce the top-down coincidence detection.

Using a simplified approach similar to Pinsky and Rinzel (1994), Larkum et al. (2004) have modeled dendrite-based gain modulation. The parameters in the model could be tuned to quantitatively reproduce the firing rate response of layer 5 pyramidal cells stimulated at the soma and the dendrites simultaneously. Larkum et al. (2004) concluded that a two-compartment model was sufficient to explain the time-averaged firing rate.

A more stringent requirement for neuron model validation, however, is to predict spike times (Keat et al., 2001; Pillow et al., 2005; Jolivet et al., 2006, 2008a,b; Gerstner and Naud, 2009). Given the low spike-time reliability of pyramidal neurons, spike time prediction is compared to the intrinsic reliability (Jolivet et al., 2006). This approach can be seen as predicting the instantaneous firing rate (Naud et al., 2011). Generalized integrate-and-fire models can predict instantaneous firing rate of layer 5 pyramidal neurons with substantial precision (Jolivet et al., 2008a; Gerstner and Naud, 2009; Naud et al., 2009) in the absence of dendritic stimulation. The question remains whether a neuron model can predict the spike times of layer 5 pyramidal neurons when both the dendrites and the soma are stimulated simultaneously.

We present a study of a simplified model of layer 5 pyramidal cells of the cortex with dendrites excitable with calcium spikes (Larkum et al., 2004, 2009). Following Larkum et al. (2004), we hypothesized that we can model the soma and its apical dendrite with two compartments, without significant loss in the accuracy of spike-timing predictions. We introduce experimentally measurable impulse-response functions (Segev et al., 1995), which transfer the effect of current injected in one compartment to current reaching the other. The impulse-response functions replace the instantaneous connection used in previous two-compartment models (Pinsky and Rinzel, 1994; Larkum et al., 2004) and acts as a third, passive, compartment. Each compartment was modeled with a pair of non-linear differential equations with a small number of parameters that approximate the Hodgkin-and-Huxley equations. The predictive power of this model was tested on electrophysiological experiments where noisy current was injected in both the soma and the apical dendrite simultaneously (Larkum et al., 2004).

## 2. Methods

Methods are separated in four parts. First we present the model, second the experimental protocol, then fitting methods and finally the analysis methods.

### 2.1. Description of the model

**Figure 1:**
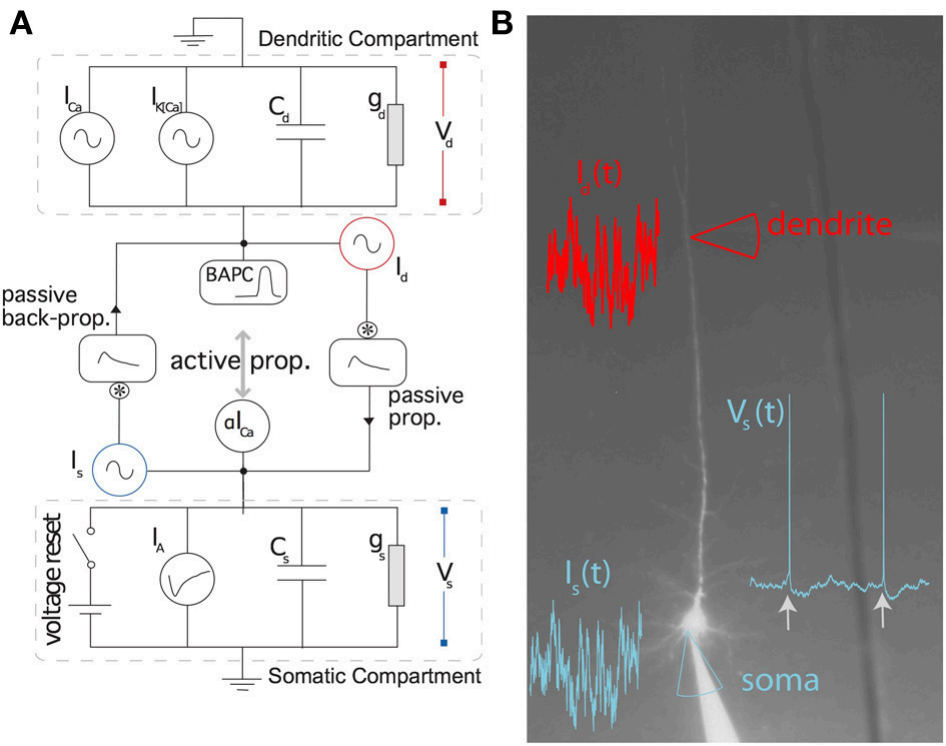
**Schematic representation of the two-compartment model. (A)** Somatic and dendritic compartment communicate through passive and active propagation. Passive communication filters through a convolution (denoted by an asterisk) the current injected in the other compartment. Active communication in the soma introduces a perturbation proportional to the dendritic current *I_Ca_*. Active communication to the dendrites introduces a stereotypical back-propagating action potential current (BAPC). The somatic compartment has spike-triggered adaptation and a moving threshold. The dendritic compartment has an activation current and recovery current. **(B)** Associated experimental protocol with current injection both in soma and apical dendrite of layer 5 pyramidal cells of the rat somato-sensory cortex. Variables are defined in the main text.

Figure 1 shows a schematic representation of the two-compartment model. In details, the model follows the system of differential equations:

(1) $$\begin{array}{l} C_{s}\frac{dV_{s}}{dt}=-g_{s}(V_{s}-E_{s})+\alpha m+I_{s}\\ \;+\sum_{\left\{{\hat{t}}_{i}\right\}}I_{A}(t-{\hat{t}}_{i})+\epsilon_{ds}*I_{d} \end{array}$$

(2) $$\begin{array}{l} C_{d}\frac{dV_{d}}{dt}=-g_{d}(V_{d}-E_{d})+g_{1}m+g_{2}x+I_{d}\\ \;+\sum_{\left\{{\hat{t}}_{i}\right\}}I_{BAP}(t-{\hat{t}}_{i})+\epsilon_{sd}*I_{s} \end{array}$$

(3) $$\tau_{m}\frac{dm}{dt}=\frac{1}{1+\exp\left(-\frac{V_{d}-E_{m}}{D_{m}}\right)}-m$$

(4) $$\tau_{x}\frac{dx}{dt}=m-x$$

(5) $$\tau_{T}\frac{dV_{T}}{dt}=-(V_{T}-E_{T})+D_{T}\sum_{\left\{{\hat{t}}_{i}\right\}}\delta (t-{\hat{t}}_{i})$$

where *I_s_* is the current injected in the soma, *I_d_* the current injected in the dendrites, *V_s_* is the somatic voltage, *V_d_* is the dendritic voltage, *m* is the level of activation of a putative calcium current (*I*_Ca_ = *g*_1*m*_), *x* is the level of activation of a putative calcium-activated potassium current (*I*_K(Ca)_ = *g*_2*x*_), *V_T_* is the dynamic threshold for firing somatic spikes, *I_A_* is a spike-triggered current mediating adaptation, *I_BAP_* is the the current associated with the back-propagating action potential, ϵ_*sd*_ is the filter relating the current injected in the soma to the current arriving in the dendrite and ϵ_*ds*_ is the filter relating the current injected in the dendrite to the current arriving in the soma. The spikes are emitted if *V_s_(t)* > *V_T_(t)* which results in $\hat{t}$_*(last)*_ = *t* while *V_s_* → *E_r_* and *t* → *t* + τ_*R*_. The parameters are listed in Table 1.

**Table 1:** **List of parameters and their fitted value for the two-compartment model**.

**Variable**		**Value**	**Units**
Somatic leak conductance	*g_s_*	22	nS
Somatic capacitance	*C_s_*	379	pF
Somatic reversal potential	*E_s_*	−73	mV
Threshold baseline	*E_T_*	−53	mV
Spike-triggered jump in threshold	*D_T_*	2.0	mV
Time-constant of dynamic threshold	τ*_T_*	27	ms
Maximum “Ca” current	*g*_1_	567	pA
Maximum effect of “Ca” current in soma	α	337	n.u.
Dendritic leak conductance	*g_d_*	22	nS
Dendritic capacitance	*C_d_*	86	pF
Dendritic reversal potential	*E_d_*	−53	mV
Time-constant for variable *m*	τ_*m*_	6.7	ms
Time-constant for variable *x*	τ_*x*_	49.9	ms
Sensitivity of “Ca” Current	*D_m_*	5.5	ms
Maximum “K(Ca)” Current	*g*_2_	−207	pA
Half-activtion potential of “Ca” current	*E_m_*	−0.6	mV

As a control, we also consider an entirely passive model of dendritic integration. In this model, the current injected in the dendrite is filtered passively to reach the soma. The generalized passive model has an instantaneous firing rate:

(6) $$\lambda (t)=\lambda_{0}\exp\left(\kappa_{s}*I_{s}+\kappa_{ds}*I_{d}+\sum_{\left\{{\hat{t}}_{i}\right\}}\eta_{A}(t-{\hat{t}}_{i})\right)$$

where λ_0_ is a constant related to the reversal potential, κ_*s*_ somatic membrane filter, κ_*ds*_ is the filter relating the current injected in the dendrite to the voltage change in the soma, and η_*A*_ is the effective spike-triggered adaptation.

### 2.2. Experimental protocol

Animal handling was in strict accordance with the guidelines given by the veterinary office of the canton Bern-Switzerland. Parasagittal brain slices of the somato-sensory cortex (300–350 m thick) were prepared from 28–35 day-old Wistar rats. Slices were cut in ice-cold extracellular solution (ACSF), incubated at 34°C for 20 min and stored at room temperature. During experiments, slices were superfused with in ACSF at 34°C. The ACSF contained (in mM) 125 NaCl, 25 NaHCO3, 25 Glucose, 3 KCl, 1.25 NaH2PO4, 2 CaCl2, 1 MgCl2, pH 7.4, and was continuously bubbled with 5% CO2/95% O2. The intracellular solution contained (in mM) 115 K+-gluconate, 20 KCl, 2 Mg-ATP, 2 Na2-ATP, 10 Na2-phosphocreatine, 0.3 GTP, 10 HEPES, 0.1, 0.01 Alexa 594 and biocytin (0.2%), pH 7.2.

Recording electrodes were pulled from thick-walled (0.25 mm) borosilicate gla-ss capillaries and used without further modification (pipette tip resistance 5–10 MΩ for soma and 20–30 MΩ for dendrites). Whole-cell voltage recordings were performed at the soma of a layer V pyramidal cell. After opening of the cellular membrane a fluorescent dye, Alexa 594 could diffuse in the entire neuron allowing to perform patch clamp recordings on the apical dendrite 600–700 μm from the soma. Both recordings were obtained using Axoclamp Dagan BVC-700A amplifiers (Dagan Corporation). Data was acquired with an ITC-16 board (Instrutech) at 10 kHz driven by routines written in the Igor software (Wavemetrics).

The injection waveform consisted of 6 blocks of 12 s. Each block is made of three parts: (1) one second of low-variance colored noise injected only in the soma, (2) one second of low-variance colored noise injected only in the dendritic injection site, (3) ten seconds of high-variance colored noise whose injection site depends on the block: In the first block, the 10-s stimulus is injected only in the dendritic site, the second block delivers the 10-s stimulus in the soma only, and the four remaining blocks deliver simultaneous injections in the soma and the dendrites. The colored noise was simulated with MATLAB as an Ornstein-Uhlenbeck process with a correlation time of 3 ms. The six blocks make a 72 s stimulus that was injected repeatedly without redrawing the colored noise (frozen-noise). Noise is frozen across repetitions to estimate intrinsic reliability, but not across blocks to ensure independent test set and training set. Twenty repetitions of the 72-s stimulus were carried out, separated by periods of 2–120 s. Out of the twenty repetitions, a set of seven successive repetitions were selected on the basis of high intrinsic reliability.

### 2.3. Fitting methods

Each kernel (κ_*s*_, κ_*ds*_, η_*A*_, ϵ_*ds*_, ϵ_*sd*_, *I_A_*, *I_BAP_*) is expressed as a linear combination of non-linear basis (i.e., *κ_s_(t) = ∑_i_a_i_f_i_(t)*). The rectangular function was chosen as the non-linear basis. The parameters weighting the contributions of the different rectangular functions are then linear in the derivative of the membrane potential for the two-compartment model and generalized linear for the passive model.

For the two-compartment model, we use a combination of regression methods and exhaustive search to maximize the mean square-error of the voltage derivative. The regression methods are similar to those previously used for estimating parameters with intracellular recordings. These methods are described in more details in Jolivet et al. (2006); Paninski et al. (2005); Mensi et al. (2012); Pozzorini et al. (2013). First, we distinguish two types of parameters, the parameters that can be expressed as a linear function of the observables and the parameters that cannot. For instance, the parameter *g_s_* is linear in the observable *dV_s_/dt* (Equation 1). Similarly, the amplitudes *a_i_* defining the filters are also linear parameters. There is a total of four non-linear parameters in the two-compartment model, namely τ_*m*_, *D_m_*, *E_m_*, τ_*x*_.

The fit of the somatic compartment essentially follows (Jolivet et al., 2006) but using multi-linear regression to fit the linear parameters. The fit of the dendritic compartment needs to iterate through the restricted set of non-linear parameters. All fits are performed only on the part of the data restricted for training the model. Each step in the fitting procedure uses the entire training set.

1. Fit of the dendritic compartment, knowing the injected currents and the somatic spiking history:
  1a: Compute the first-order estimate of *dV_d_/dt*;
  1b: Find the best estimates of the dendritic parameters linear in *dV_d_/dt* given a set of non-linear parameters (τ_*m*_, *D_m_*, *E_m_*, τ_*x*_). The best estimates are chosen through multi-linear regression to minimize the mean square error of *dV_d_/dt*.
  1c: Compute iteratively step 1b on a grid of the non-linear parameters (τ_*m*_, *D_m_*, *E_m_*, τ_*x*_) and find the non-linear parameters that yield the minimum mean square error of *dV_d_/dt*.
2. Fit of the somatic compartment using the fitted dendritic compartment.
  2a: Compute the first-order estimate of *dV_s_/dt*.
  2b: Find the best estimates of the somatic parameters linear in *dV_s_/dt* given a set of non-linear parameters (*D_T_*, τ_*T*_, *E_T_*). The best estimates are chosen through linear regression to minimize the mean square error of *dV_s_/dt*.
  2c: Compute iteratively step 2b on a grid of the non-linear parameters and simulate the model with each set of non-linear parameters in order to compute the coincidence rate Γ (see Section 2.4).
  2d: Take the parameters that yield the maximum Γ coincidence factor.

For the generalized linear model, we use maximum likelihood methods (Paninski, 2004; Pillow et al., 2005). Expressing the kernels as a linear combination of rectangular bases we recover the generalized linear model. Here the link-function is exponential so that the likelihood is convex. We therefore performed a gradient ascent of the likelihood to arrive at the optimal parameters.

### 2.4. Analysis methods

When one focuses on spike timing, one may want to apply methods that compare spike trains in terms of a spike-train metric (Victor and Purpura, 1996) or the coincidence rate (Kistler et al., 1997). Both measures can be used to compare a recorded spike train with a model spike train. A model which achieve an optimal match in terms of spike-train metrics will automatically account for global features of the spike train such as the interspike interval distribution.

Here we used the averaged coincidence rate Γ (Kistler et al., 1997). The coincidence rate, like most other spike time metrics, can be related to the coefficient of correlation between the instantaneous firing rate of the model and the neuron (Naud et al., 2011). It can be seen as a similarity measure between pairs of spike trains, averaged on all possible pairs. To compute the pairwise coincidence rate, one first finds the number of spikes from the model that fall within an interval of Δ = 4 ms after or before a spike from the real neuron. This is called the number of coincident events *N_nm_* between neuron repetition *n* and model repetition *m*. The coincidence rate is the ratio of the number of coincident events over the averaged number of events 0.5(*N_n_* + *N_m_*), where *N_n_* is the number of spikes in the neuron spike train and *N_m_* is the number of spikes in the model spike train. This ratio is then scaled by the number of chance coincidences *N*_Poisson_ = 2Δ*N_m_N_n_/T*. This formula comes from the number of expected coincidences assuming a Poisson model at a fixed rate *N_m_/T* where *T* is the time length of each individual spike trains. The scaled coincidence rate is

(7) $$\Gamma_{nm}=\frac{N_{nm}-N_{\text{Poisson}}}{0.5(1-N_{\text{Poisson}}/N_{n})(N_{n}+N_{m})}.$$

The pairwise coincidence rate Γ_*nm*_ is then averaged across all possible pairings of spike trains (trials) generated from the model with those from the neuron and gives the averaged coincidence rate Γ. Averaging across all possible pairings of spike trains from the neuron with a distinct repetition of the same stimulus given to the same neuron gives the intrinsic reliability *R*.

## 3. Results

Dual patch-clamp recordings were performed in L5 Pyramidal cells of Wistar rats (see Experimental Methods). A simplified two-compartment model (see Model Description) was fitted on the first 36 s of stimulation for all repetitions. The rest of the data (36 s) was reserved to evaluate the model's predictive power. The predictive power of the two-compartment model with active dendrites was then compared to a model without activity in the dendrites (see Section 2.1), the generalized linear passive model.

**Figure 2:**
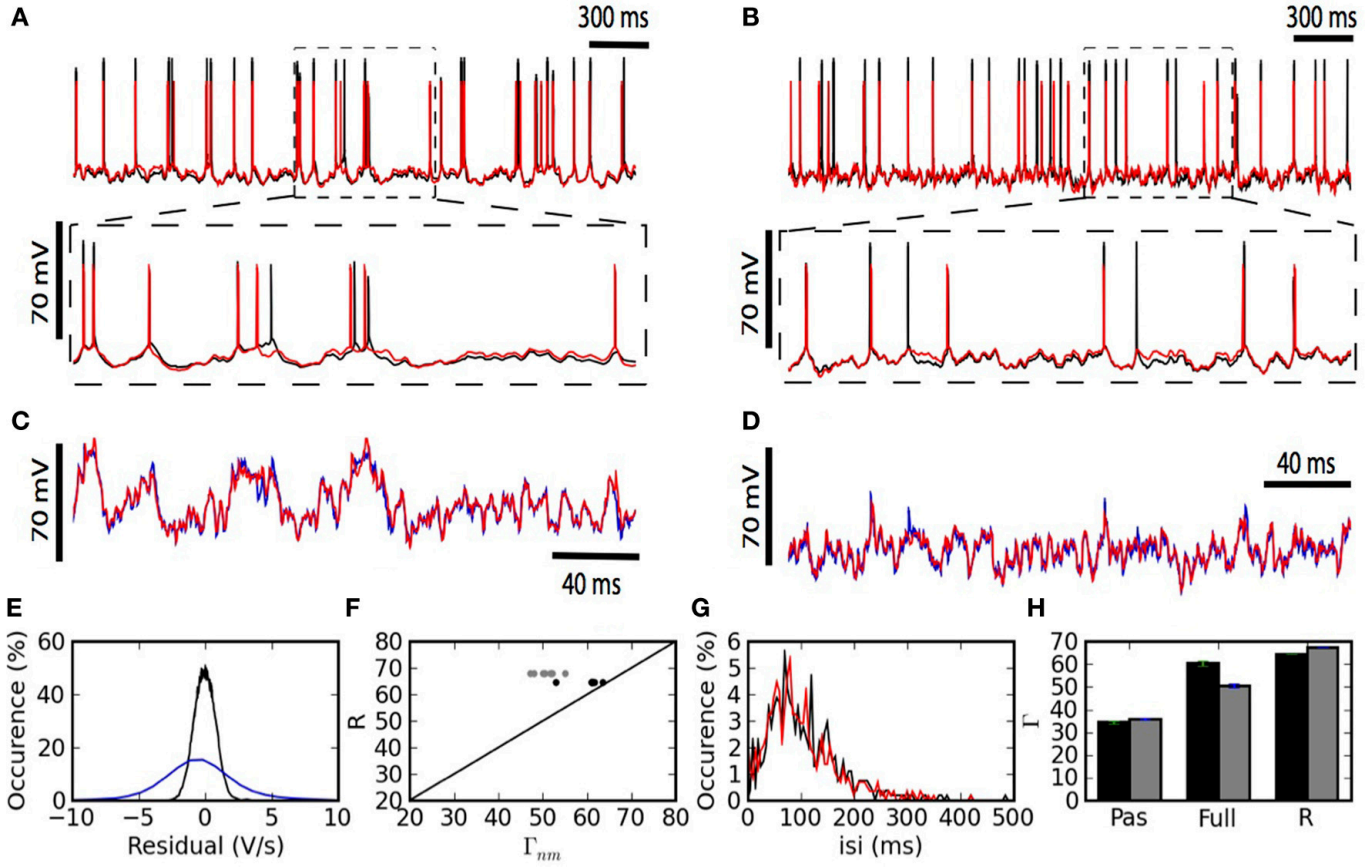
**The two-compartment model fits qualitatively and quantitatively the electrophysiological recordings. (A,B)** Overlay of the model (red) and experimental (black) somatic voltage trace. The dashed box indicates an area stretched out for higher precision. **(C,D)** The overlay of model (red) and experimental (blue) dendritic voltage is shown for the stretched sections in **(A,B)**. Left **(A,C)** and right **(B,D)** columns show two different injection regimes contrasting by the amount of dendritic activity which is high for **(A,C)** and medium for **(B,D)**. **(E)** Residuals from the linear regression are shown for the somatic (black) and dendritic (blue) compartment. **(F)** For each repetition the Γ Coincidence factor is plotted against the intrinsic reliability of the cell. Gray points show the performance of the model on the test set and black points show the performance of the model on the training set. **(G)** Comparison of the inter-spike interval histogram for the model (red) and the experiment (black). **(H)** Comparison of the generalized passive (Pas), and the full two-compartment model (Full) with the intrinsic reliability (R) of the neuron in terms of the Γ coincidence factor. The averaged Γ factor is shown for the training set (black) and test set (Gray).

Figure 2 summarizes the predictive power of the two-compartment model. The somatic and dendritic voltage traces are well captured (Figures 2A–D). The main cause for erroneous prediction of the somatic voltage trace is extra or missed spikes (Figures 2A,B lower panels). The dendritic voltage trace of the model follows the recorded trace both in a low dendritic-input regime (Figure 2C) and in a high dendritic-input regime with dendritic “spikes” (Figure 2D). The greater spread of voltage-prediction-error (Figure 2) is mainly explained by the larger range of voltages in the dendrites (somatic voltage prediction is strictly subthreshold whereas dendritic voltage prediction ranges from −70 to + 40 mV). The interspike interval distribution is well predicted by the model (Figure 2G).

The generalized passive model does not predict as many spike times (Figure 2H). The intrinsic variability in the test set was 68% and the two-compartment model predicted 50%. The prediction falls to 36% in the absence of a dendritic non-linearity (Figure 2H).

**Figure 3:**
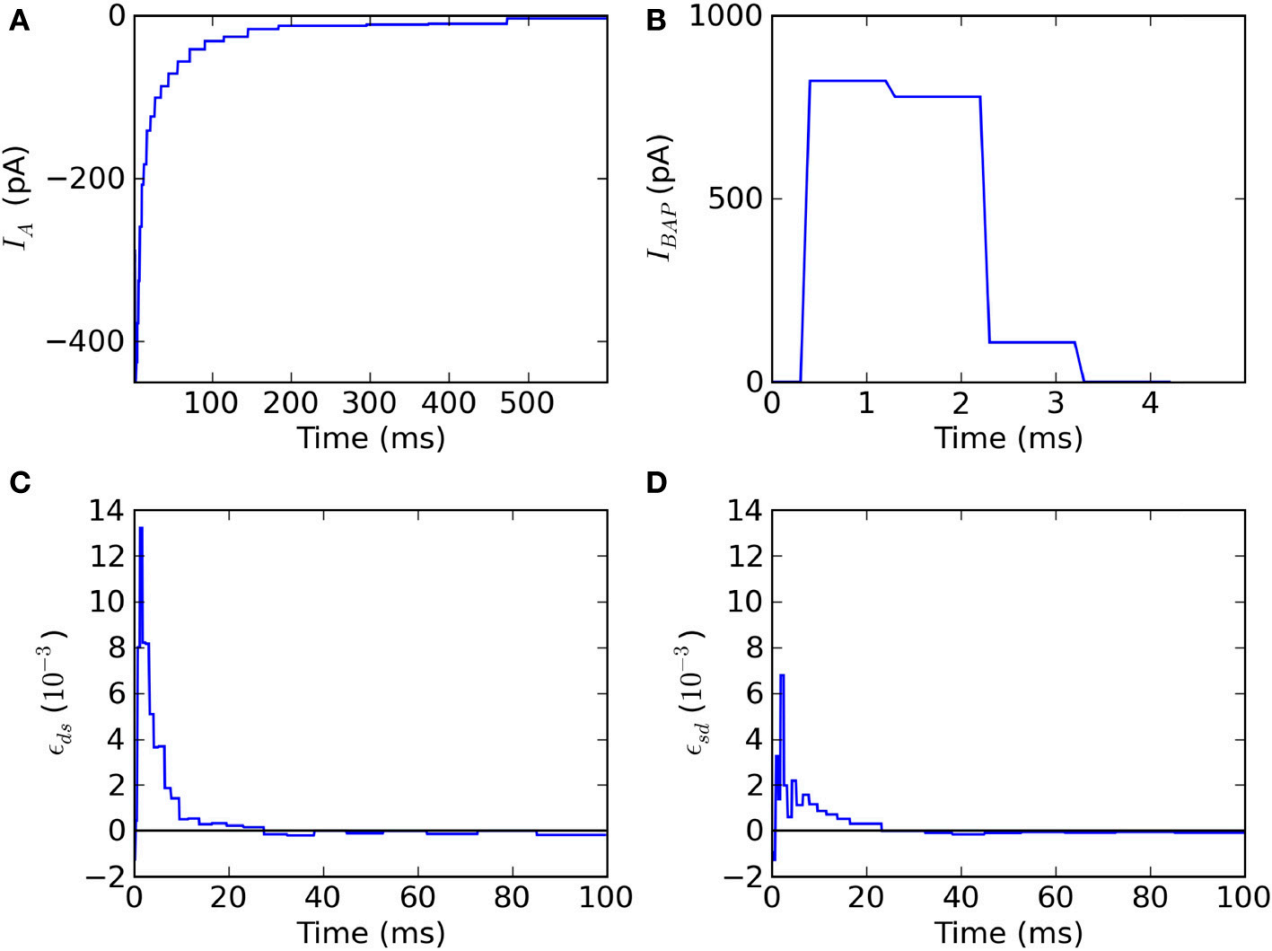
**Fitted kernels of the two-compartment model. (A)** The kernel *I_A_(t)* for spike-triggered adaptation is negative and increases monotonically between 6 and 600 ms. **(B)** The back-propagating current *I*_BAP_(*t*)reaching the dendrites is a short (2 ms) and strong (900 pA) pulse. **(C)** The convolution kernel ϵ*_ds_(t)* linking the current injected in the dendrite to the current reaching the soma. **(D)** The convolution kernel ϵ*_sd_(t)* linking the current injected in the soma to the current reaching the dendrite.

The fitted kernels show that spike triggered adaptation is a monotonically decaying current that starts very strongly and decays slowly for at least 500 ms (Figure 3A). The back-propagating action potential is mediated by a strong pulse of current lasting 2–3 ms (Figure 3B). The coupling ϵ_*ds*_ from dendrite to soma has a maximal response after 2–3 ms and then decays so as to be slightly negative after 35 ms (Figure 3C). The coupling ϵ_*sd*_ from soma to dendrite follows qualitatively ϵ_*ds*_ with smaller amplitudes and slightly larger delays for the maximum and minimum peaks (Figure 3D), consistent with the larger membrane time-constant in the soma than in the dendrites.

**Figure 4:**
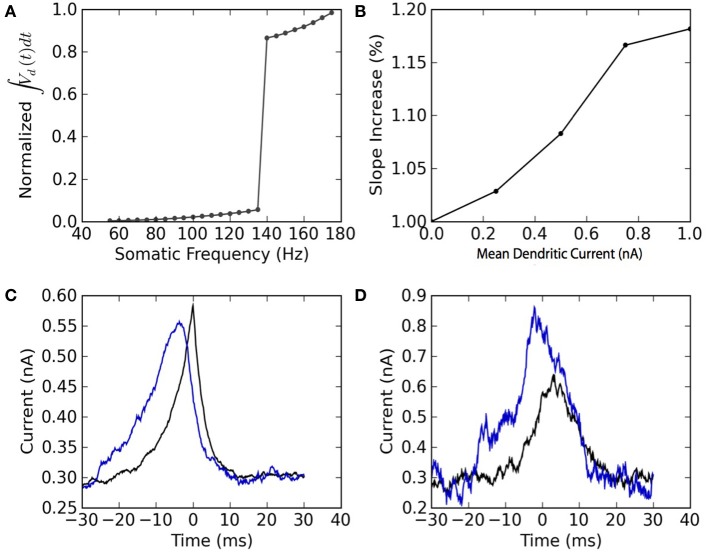
**The model reproduces the qualitative features of active dendrites reported in Larkum et al. (1999) and Larkum et al. (2004). (A)** Dendritic non-linearity is triggered by somatic spiking above a critical frequency. Somatic spike-trains of 5 spikes are forced in the soma of the mathematical model at different firing frequencies. The normalized integral of the dendritic voltage is shown as a function of the somatic spiking frequency. **(B)** Dendritic injection modulates the slope of the somatic spiking-frequency vs. current curve. The slope of the frequency vs mean somatic current as measured between 5 and 50 Hz is plotted as a function of the mean dendritic current. Both somatic and dendritic currents injected are Ornstein-Uhlenbeck processes with a correlation time of 3 ms and a standard deviation of 300 pA. **(C)** Spike-triggered average of the current injected in the soma (black) and in the dendrites (blue). **(D)** Burst-triggered average of the current injected in the soma (black) and in the dendrites (blue). The fact that the blue curve is higher than the black curve, and that this relation is inverted in **(C)**, may be interpreted as a top-down coincidence detection by bursts.

The two-compartment model can reproduce qualitative features associated with the dendritic non-linearity in the apical dendrite of L5 pyramidal neurons. We study two of these features: the critical frequency (Larkum et al., 1999) and the gain modulation (Larkum et al., 2004). The first relates to the critical somatic firing frequency above which a non-linear response is seen in the soma, reflecting calcium channel activation in the dendrites. To simulate the original experiment, we force 5 spikes in the soma at different frequencies and plot the integral of the dendritic voltage. The critical frequency for initiating a non-linear increase in summed dendritic voltage is 138 Hz (Figure 4A). Pérez-Garci et al. (2006) reported a critical frequency of 105 Hz while (Larkum et al., 1999) reported 85 Hz. This appears to vary across different cells and pharmacological conditions.

The model also appears to perform gain modulation as in Larkum et al. (2004) (Figure 4B). The relation between somatic firing rate and mean somatic current depends on the dendritic excitability. The firing threshold but also the gain (or slope) of the somatic frequency vs. somatic current curve depend on the mean dendritic current. The gain modulation is attributed to a greater presence of bursts (Figure 4B) caused by dendritic calcium-current activation at higher dendritic input. The link between burst and dendritic activity is reflected in the burst- and spike-triggered average injected current (Figures 4C,D) similar to Larkum et al. (2004). The burst-triggered current is greater for the dendritic injection, whereas the spike-triggered current is larger for somatic injection. Therefore bursts signal a higher dendritic current that was concomittant with an increased somatic current. This can be interpreted as a top-down coincidence detection.

## 4. Discussion

A dendrite is said active when it sustains either sodium, calcium or NMDA spikes. Our model reflects calcium spikes in the dendritic compartment, but not dendritic sodium spikes or NMDA spikes. The parameters fitted (Table 1) are in agreement with voltage-activated calcium channels. An activation sensitivity *D_m_* of 5 mV is typical of many ion channels, and the time constant τ_*x*_ of about 50 ms is slower than the high-voltage activated calcium channel which has a time constant of about 10 ms (Gerstner et al., 2014). The current injection in the apical dendrite presumably does not solicit NMDA spikes known to occur in the apical tuft (Larkum et al., 2009).

Even if the spike-time prediction is high, the fitted parameters may differ from the real biophyical parameters for various reasons. First, the fitting method we used avoids local minima combining convex fitting procedures with exhaustive search. Even if the steps in the procedure are convex, the sequence of such steps may not be convex. Therefore the fitted parameters may reflect a local minimum. Also, the drop in coincidence rate between the training set and the test set indicate that overfitting is present. This could be avoided by using a smaller number of non-linear bases for the filters, or the use of raised cosine functions instead of the rectangular ones. Lastly, the filters and reduced model parameters may lump together different biophysical processes. The particular shape of the filter will depend on the average membrane potential and the average firing rate. This is one reason why we did not estimate the filters empirically with a separate set of experiments, but instead we fitted the model on current injection designed to imitate the natural condition.

## 5. Conclusion

Using a two-compartment model interconnected with temporal filters, we were able to predict a substantial fraction of spike times. The predicted spike trains achieved an averaged coincidence rate of 50%. The scaled coincidence rate obtained by dividing by the intrinsic reliability (Jolivet et al., 2008a; Naud and Gerstner, 2012) was 72%, which is comparable to the state-of-the performance for purely somatic current injection which reaches up to 76% (Naud et al., 2009). Comparing with a passive model for dendritic current integration, we found that the predictive power decreased to a scaled coincidence rate of 53%. Therefore we conclude that regenerating activity in the apical dendrite is required to properly account for the dynamics of layer 5 pyramidal cells under *in-vivo*-like conditions.

### Conflict of interest statement

The authors declare that the research was conducted in the absence of any commercial or financial relationships that could be construed as a potential conflict of interest.

## References

[B1] Archie K. Mel B. (2000). A model for intradendritic computation of binocular disparity. Nat. Neurosci. 3, 54–63 10.1038/71125 10607395

[B2] Branco T. Clark B. A. Michael H. (2010). Dendritic discrimination of temporal input sequences in cortical neurons. Science 329, 1671–1675 10.1126/science.1189664 20705816 PMC6354899

[B3] Gabbiani F. Krapp H. G. Koch C. Laurent G. (2002). Multiplicative computation in a visual neuron sensitive to looming. Nature 420, 320–324 10.1038/nature01190 12447440

[B4] Gerstner W. Kistler W. Naud R. Paninski L. (2014). Neuronal Dynamics. Cambridge, UK: Cambridge University Press

[B5] Gerstner W. Naud R. (2009). How good are neuron models? Science 326, 379–380 10.1126/science.1181936 19833951

[B6] Golding N. L. Spruston N. (1988). Dendritic sodium spikes are variable triggers of axonal action potentials in hippocampal ca1 pyramidal neurons. Neuron 21, 1189–1200 10.1016/S0896-6273(00)80635-2 9856473

[B7] Jolivet R. Kobayashi R. Rauch A. Naud R. Shinomoto S. Gerstner W. (2008a). A benchmark test for a quantitative assessment of simple neuron models. J. Neurosci. Methods 169, 417–424 10.1016/j.jneumeth.2007.11.006 18160135

[B8] Jolivet R. Rauch A. Lüscher H. Gerstner W. (2006). Predicting spike timing of neocortical pyramidal neurons by simple threshold models. J. Comput. Neurosci. 21, 35–49 10.1007/s10827-006-7074-5 16633938

[B9] Jolivet R. Schürmann F. Berger T. K. Naud R. Gerstner W. Roth A. (2008b). The quantitative single-neuron modeling competition. Biol. Cybern. 99, 417–426 10.1007/s00422-008-0261-x 19011928

[B10] Keat J. Reinagel P. Reid R. C. Meister M. (2001). Predicting every spike a model for the responses of visual neurons. Neuron 30, 803–817 10.1016/S0896-6273(01)00322-1 11430813

[B11] Kistler W. Gerstner W. Hemmen J. (1997). Reduction of the hodgkin-huxley equations to a single-variable threshold model. Neural Comput. 9, 1015–1045 10.1162/neco.1997.9.5.1015

[B12] Larkum M. E. Nevian T. Sandler M. Polsky A. Schiller J. (2009). Synaptic integration in tuft dendrites of layer 5 pyramidal neurons: a new unifying principle. Science 325, 756–760 10.1126/science.1171958 19661433

[B13] Larkum M. E. Senn W. Luscher H. R. (2004). Top-down dendritic input increases the gain of layer 5 pyramidal neurons. Cereb. Cortex 14, 1059–1070 10.1093/cercor/bhh065 15115747

[B14] Larkum M. E. Zhu J. Sakmann B. (1999). A new cellular mechanism for coupling inputs arriving at different cortical layers. Nature 398, 338–341 10.1038/18686 10192334

[B15] Larkum M. E. Zhu J. J. Sakmann B. (2001). Dendritic mechanisms underlying the coupling of the dendritic with the axonal action potential initiation zone of adult rat layer 5 pyramidal neurons. J. Physiol. (Lond.) 533, 447–466 10.1111/j.1469-7793.2001.0447a.x 11389204 PMC2278642

[B16] Legenstein R. Maass W. (2011). Branch-specific plasticity enables self-organization of nonlinear computation in single neurons. J. Neurosci. 31, 10787–10802 10.1523/JNEUROSCI.5684-10.2011 21795531 PMC6623094

[B17] Llinas R. Sugimori M. (1980). Electrophysiological properties of *in vitro* purkinje cell dendrites in mammalian cerebellar slices. J. Physiol. 305, 197–213 7441553 10.1113/jphysiol.1980.sp013358 PMC1282967

[B18] Mensi S. Naud R. Avermann M. Petersen C. C. H. Gerstner W. (2012). Parameter extraction and classification of three neuron types reveals two different adaptation mechanisms. J. Neurophysiol. 107, 1756–1775 10.1152/jn.00408.2011 22157113

[B19] Naud R. Berger T. Bathellier B. Carandini M. Gerstner W. (2009). Quantitative single-neuron modeling: competition 2009. Front. Neur. Conference Abstract: Neuroinformatics 2009. 10.3389/conf.neuro.11.2009.08.10620224819

[B20] Naud R. Gerhard F. Mensi S. Gerstner W. (2011). Improved similarity measures for small sets of spike trains. Neural Comput. 23, 3016–3069 10.1162/NECO-a-00208 21919785

[B21] Naud R. Gerstner W. (2012). Spike Timing: Mechanisms and Function, Chapter Can We Predict Every Spike. Boca Raton, FL: CRC Press

[B22] Paninski L. (2004). Maximum likelihood estimation of cascade point-process neural encoding models. Network 15, 243–262 10.1088/0954-898X/15/4/002 15600233

[B23] Paninski L. Pillow J. W. Simoncelli E. (2005). Comparing integrate-and-fire models estimated using intracellular and extracellular data. Neurocomputing 65–66, 379–385 10.1016/j.neucom.2004.10.032

[B24] Pérez-Garci E. Gassmann M. Bettler B. Larkum M. E. (2006). The gabab1b isoform mediates long-lasting inhibition of dendritic ca2+ spikes in layer 5 somatosensory pyramidal neurons. Neuron 50, 603–616 10.1016/j.neuron.2006.04.019 16701210

[B25] Pillow J. W. Paninski L. Uzzell V. J. Simoncelli E. P. Chichilnisky E. J. (2005). Prediction and decoding of retinal ganglion cell responses with a probabilistic spiking model. J. Neurosci. 25, 11003–11013 10.1523/JNEUROSCI.3305-05.2005 16306413 PMC6725882

[B26] Pinsky P. Rinzel J. (1994). Intrinsic and network rhythmogenesis in a reduced traub model for ca3 neurons. J. Comput. Neurosci. 1, 39–60 10.1007/BF00962717 8792224

[B27] Polsky A. Mel B. Schiller J. (2004). Computational subunits in thin dendrites of pyramidal cells. Nat. Neurosci. 7, 621–627 10.1038/nn1253 15156147

[B28] Pozzorini C. Naud R. Mensi S. Gerstner W. (2013). Temporal whitening by power-law adaptation in neocortical neurons. Nat. Neurosci. 16, 942–948 10.1038/nn.3431 23749146

[B29] Schaefer A. Larkum M. E. Sakmann B. Roth A. (2003). Coincidence detection in pyramidal neurons is tuned by their dendritic branching pattern. J. Neurophysiol. 89, 3143–3154 10.1152/jn.00046.2003 12612010

[B30] Schiller J. Major G. Koester H. J. Schiller Y. (2000). Nmda spikes in basal dendrites of cortical pyramidal neurons. Nature 404, 285–289 10.1038/35005094 10749211

[B31] Segev I. Rall W. Rinzel J. (1995). The Theoretical Foundation of Dendritic Function. Cambridge, MA: MIT Press

[B32] Stuart G. Spruston N. Häusser M. (2007). Dendrites, 2nd Edn. Oxford: Oxford University Press ISBN: 9780198566564 (alk. paper). 10.1093/acprof:oso/9780198566564.001.0001

[B33] Taylor W. R. He S. Levick W. R. Vaney D. I. (2000). Dendritic computation of direction selectivity by retinal ganglion cells. Science 289, 2347–2350 10.1126/science.289.5488.2347 11009420

[B34] Traub R. D. Wong R. K. S. Miles R. Michelson H. (1991). A model of a CA3 hippocampal pyramidal neuron incorporating voltage-clamp data on intrinsic conductances. J. Neurophysiol. 66, 635–650 1663538 10.1152/jn.1991.66.2.635

[B35] Victor J. D. Purpura K. (1996). Nature and precision of temporal coding in visual cortex: a metric-space analysis. J. Neurophysiol. 76, 1310–1326 8871238 10.1152/jn.1996.76.2.1310

